# Perception of risk regarding the use of COVID-19 vaccine among pregnant women in Motta town and Hulet Eji Enese district, northwest Ethiopia

**DOI:** 10.1371/journal.pone.0269591

**Published:** 2022-08-24

**Authors:** Melaku Hunie Asratie, Belayneh Ayanaw Kassie, Daniel Gashaneh Belay, Mastewal Endalew, Moges Gashaw, Getnet Melak Assegie

**Affiliations:** 1 Department of Women’s and Family Health, School of Midwifery, College of Medicine and Health sciences, University of Gondar, Gondar, Ethiopia; 2 Department of Human Anatomy, College of medicine and Health Sciences, University of Gondar, Gondar, Ethiopia; 3 Department of Epidemiology and Biostatistics, Institute of Public Health, College of Medicine and Health Sciences, University of Gondar, Gondar, Ethiopia; 4 Department of Environmental and Occupational Health, and Safety, Institute of Public Health, College of Medicine and Health sciences, University of Gondar, Gondar, Ethiopia; 5 Department of Physiotherapy, School of Medicine, College of Medicine and Health sciences, University of Gondar, Gondar, Ethiopia; 6 Department of Economics, School of Economics and Management, University of Ferrara, Ferrara, Italy; Institute of Science, Banaras Hindu University, Varanasi, INDIA

## Abstract

**Background:**

COVID-19 vaccination during pregnancy is a common practice in developing countries like Ethiopia. Despite there being a rumor from the community that the use of COVID-19 vaccination during pregnancy is associated with many pregnancy adverse outcomes. However, there is a paucity of empirical evidence on the perception of risk COVID-19 vaccination during pregnancy in Ethiopia. This study assessed the perception of risk COVID-19 vaccination during pregnancy and associated factors in Motta town and Hulet Eji Enese district, northwest Ethiopia.

**Methods:**

A community-based cross-sectional study was conducted from December 12 to February 12, 2021. A total of 851 women’ were selected using the stratified cluster sampling technique. Data were collected by face-to-face interview using a semi-structured pretested and interviewer-administered questionnaire. A multivariable logistic regression model was fitted to identify factors associated with the perception of risk COVID-19 vaccination during pregnancy. The adjusted odds ratio (AOR) with a 95% confidence interval at a p-value of ≤ 0.05 was used to declare the level of significance.

**Results:**

Perception of risk COVID-19 vaccination during pregnancy was 34.2% (95%CI (Confidence Interval): 31–37). Unplanned pregnancy (AOR = 3.66; 95%CI: 2.31–5.81), long travel time to the nearby health care facility (AOR: 4.57; 95% CI: 2.34–8.91), have no formal education (AOR: 3.15; 95%CI: 1.71–5.79), attending secondary educational level (AOR: 5.18; 95% CI: 2.17–12.4), no ANC (Antenatal Care) service utilization (AOR: 7.07; 95% CI: 4.35–11.5) and negative attitude towards COVID-19 vaccination (AOR: 6.05; 95%CI: 3.88–9.43) were significantly associated with the perception of risk COVID-19 vaccination during pregnancy.

**Conclusions:**

Most of the participants perceive COVID-19 vaccination during pregnancy as a risk for the outcome of pregnancy. Designing strategies to increase women’s educational status, promoting the need for maternal and child health services, and awareness creation regarding COVID-19 vaccination will have a great role in changing the perception of pregnant women. Therefore, the government should design public health programs targeting the identified factor, and should minimize the perception of risk acquiring infection from COVID-19 vaccine to improve maternal and neonatal health outcome.

## Background

Currently, the successful COVID-19 vaccine has originated for the general population including those pregnant women [1]. Acceptance of COVID-19 vaccination among pregnant women is determinative for the reduction of COVID-19 related morbidity and mortality [2]. Nowadays world health organizations had tried to make the COVID-19 vaccine easily available for the general population including those pregnant women [3].

Medications during pregnancy will have adverse pregnancy complications [4]. Due to this scientific mythology, pregnant women are not volunteering to use the COVID-19 vaccine. There is a research question that needs covered timely “whether the magnitude of pregnant women who had perceived COVID-19 vaccination during pregnancy is a risk high or low and what are the factors associated with the perception of risk COVID-19 vaccination during pregnancy”?

Pregnancy is a unique period in a women’s life as many changes are happening to the body system that can affect the mechanism of action of any medication administered to her [4,5]. During pregnancy, the glomerular filtration increased due to increase renal blood flow which may affect the renal clearance of medications [6]. Additionally, when women become pregnant the gastric PH becomes increased and gastric motility becomes reduced this, in turn, affects the extent of medication absorption. There is also an increase in progesterone estradiol level which can affect the hepatic metabolism of some medications [7]. There is no finding yet that shows the condition that has been there with COVID-19 vaccination.

Uptake of the COVID-19 vaccine by the pregnant women mostly they considered as a teratogenic for the fetus in utero [8,9]. There is evidence that shows medications administered to pregnant women have the potential to affect the growing fetus and pregnant women consider that the COVID-19 vaccine has also effects [10]. This perception of risk towards COVID-19 vaccination during pregnancy varies from country to country [11,12]. There is a study from total survey responses of 16 countries Vaccination campaigns for pregnant women and children should be specific for each country in order to attain the larger impact [13].

Perception of risk acquiring COVID-19 from vaccination and perception of risk of adverse pregnancy outcome from COVID-19 vaccination among pregnant women determines the acceptance of the vaccine [5,14]. Researches showed that COVID-19 vaccine acceptance among pregnant women is too low [2,15], but the reason for this low acceptance rate of COVID-19 vaccination among pregnant women is not yet studied. It is obvious that participating a pregnant woman for any type of vaccination she perceives as there is a risk for the outcome of pregnancy [16]. The perception of risk pregnant women regarding the use of COVID-19 vaccination should be studied. There is no study yet done about the perception of risk regarding the use of COVID-19 vaccination during pregnancy. Therefore, this study aimed to assess the perception of risk regarding the use of COVID-19 vaccination during pregnancy among pregnant women in Motta town and Hulet Eji Enese District, northwest Ethiopia.

## Methods

### Study design, area, and period

A community-based cross-sectional study was conducted from December 12 to February 12/2021. The study was conducted in Motta town and Hulet Eji Enese district. Currently, there are 6 kebeles in Motta town and 30 kebeles in Hulet Eji Enese district. Motta town has 1 hospital,1 health center,5 private clinics, and 4health posts whereas Hulet Eji Enese district has 8 health centers,30 health posts, and 3 private clinics. Based on the data reported from the Motta town health office the total population was around 31,483 of those 15,619 were male and 15,864 females. Whereas Hulet Eji Enese district 244,155 of those 121,078 were men and 123,077females. From the report of the Motta town health office, there were 500 Pregnant women with the first visit of antenatal care,260 women delivered in the health facility and 520 women had post-partum care (both health facility delivery and home delivery).On the other hand, two monthly reports of Hulet Eji Enese district health office were 2288 pregnant women with the first visit, 570Women delivered in the health facility, and 480 women with post-partum care (both health facility delivery and home delivery).

### Sample size determination and sampling technique

The sample size was determined by the single population proportion formula n = $\frac{\left(Za∕2)2\;x\;P(1-P\right)}{(W)2}$ estimated magnitude of perception of risk COVID-19 vaccination among pregnant women was considered to be 30 [15]. With a 5% margin of error and by assuming 2 design effects and a 10% non-response rate the final required minimum sample size was estimated to be 711. From Motta town 3 kebeles and from Hulet Eji Enese district 7 kebeles totally 10 cluster kebeles were selected by simple random sampling technique. Then from each selected 10 cluster kebeles, all pregnant women had been taken as study participants. Finally, we attain 851 study participants.

### Data collection method and procedure

The data collection tool was developed by reviewing related kinds of literature [15,17,18]. A semi-structured interviewer-administered questionnaire was used to collect the data through face-to-face interviews. Socio-demographic characteristics, reproductive and maternal health service-related characteristics, and knowledge and attitude-related characteristics were incorporated in the questionnaire. The household wealth index was used to assess the economic status of participants. Eight diploma and two BSc midwives were recruited for data collection and supervision respectively. Since we were in the era of coronavirus disease-19 (COVID 19), personal protective equipment (mask & sanitizer) was provided for the data collectors and supervisors. In addition, they were oriented to keep their distance at the time of the interview.

### Data quality control

The questionnaire was first prepared in English and translated to the local language Amharic and back to keep its consistency. Before the actual data collection, a pretest was done on 5% (43) of the sample size. Two days of training were given to check the response, language clarity, interview technique, how to keep the information, and better understanding of the overall process of data collection. During the actual data collection period, a questionnaire was checked for completeness daily by supervisors.

### Data processing and analysis

Data were checked, coded, and entered to EPI data version 3.1; then it was exported to SPSS (Statistical Package for Social Science) version 25 analysis software. Before analysis, missing values, outliers, and inconsistent data were cleared. Principal component analysis was done to analyze the wealth index. Descriptive statistics like percentage, frequency, means, standard deviation, tables, and graphs were used to present the characteristics of the study participants. The binary logistic regression model was fitted to identify the associated factors for the perception of risk COVID-19 vaccination during pregnancy. Variables with univariate p-values < = 0.2 were considered as candidate predictors of risk in the use of COVID-19 vaccination in the multivariate model. After that, all explanatory variables having a p-value of ≤ 0.2 in the bivariable analysis were entered into a multivariable logistic regression analysis to minimize the effect of possible confounding factors and to identify independent factors associated with the perception of risk COVID-19 vaccination during pregnancy. P-values < = 0.05 were considered statistically significant.

### Ethics approval and consent to participate

Ethical clearance was obtained from the Institutional Review Board (IRB) of the University of Gondar. Protective equipment like a face mask was given to data collectors and supervisors. Participants were informed clearly about the purpose and benefit of the study and written and signed informed consent was obtained from them with data collectors who wear a face mask and keep his/her distance. Those who signed written consent were only participated in the study and left those who were not volunteers to participate and consider as non-response. The confidentiality of responses was maintained throughout the research process by giving codes for participants. Personal privacy and cultural norms were respected. All consent form was translated into and administered in Amharic.

## Results

### Socio-demographic characteristics of study participants

In this study, a total of 851 pregnant women were included, with a response rate of 100%. The participant’s mean age was 31.2 years (SD ±5.76 years) and almost half 440(51.7%) of the participants were within the age group of ≥31 years old. Few of the participants 123(14.5%) were from urban areas. Almost all of the participants were orthodox by religion and 571(67.1%) of the study participants had no formal education “**Table 1**”.

**Table 1:** Socio-demographic characteristics of study participants in Motta town and Hulet Eji Enese district, Northwest Ethiopia, 2021(n = 851).

Characteristics	Frequency	Percent
**Age**		
≤20	12	1.4
21–25	274	32.2
26–30	125	14.7
≥31	440	51.7
**Wealth quintile**		
Lowest	169	19.9
Second	171	20.1
Middle	170	20
Fourth	170	20
**Religion**		
Orthodox	845	99.3
Others*	6	0.7
**Residence**		
Urban	123	14.5
Rural	728	85.5
**Educational status of women**		
No formal education	571	67.1
Grade 1–8	135	15.9
Grade 9–12	71	8.3
College and above	74	8.7
**Occupational status of women**		
Farmer	686	80.6
Government employee	47	5.5
Housewife	64	7.5
Merchant	42	5
Others**	12	1.4

Others* = Muslim &protestant, others** = Student & private employee.

### Maternal health care service and obstetrical related characteristics of participants in Motta town and Hulet Eji Enese district, northwest Ethiopia, 2021

Among 851participants 371(43.6%) of respondents were gravida 3–4, 651(67.3%) of the participants the pregnancy was planned, 408(47.9%) of the participants were Para 1–2 and 800(94%) of the participants were have no bad obstetric history. Among all study participants, 583(68.5%) of them had utilized antenatal care service and 713(83.8%) of them had exposure to herbal medicine during their pregnancy state “**Table 2**”.

**Table 2:** Obstetrical and maternal health care service related characteristics of respondents in Motta town and Hulet Eji Enese district, northwest Ethiopia, 2021.

Characteristics	Frequency	Percent
**Gravida**		
1–2	273	32.1
3–4	371	43.6
≥5	207	24.3
**Type of pregnancy**		
Unplanned	200	32.7
Planned	651	67.3
**Parity**		
1–2	408	47.9
3–4	278	32.7
≥5	165	19.4
**Bad obstetric history**		
No	800	94
Yes	51	6
**Type of bad obstetric history(n = 51)**		
Still birth	21	41.2
Abortion	20	39.2
Others*	10	19.6
**Antenatal care utilization**		
No	268	31.5
Yes	583	68.5
**Number of antenatal care visit(n = 583)**		
1–2	332	57
3&above	251	43
**Exposure to herbal medicines during pregnancy**		
No	713	83.8
Yes	138	16.2
**Distance from the nearby health facility**		
Can be reached within 30 minutes	313	36.7
It takes 30 minutes to one hr.	203	23.9
It takes one hr. and above	335	39.4

Others* = disabled baby and early neonatal loss.

### Knowledge and attitude related to COVID-19 vaccination during pregnancy among pregnant women in Motta town and Hulet Eji Enese district, northwest Ethiopia, 2021

**Fig 1:**
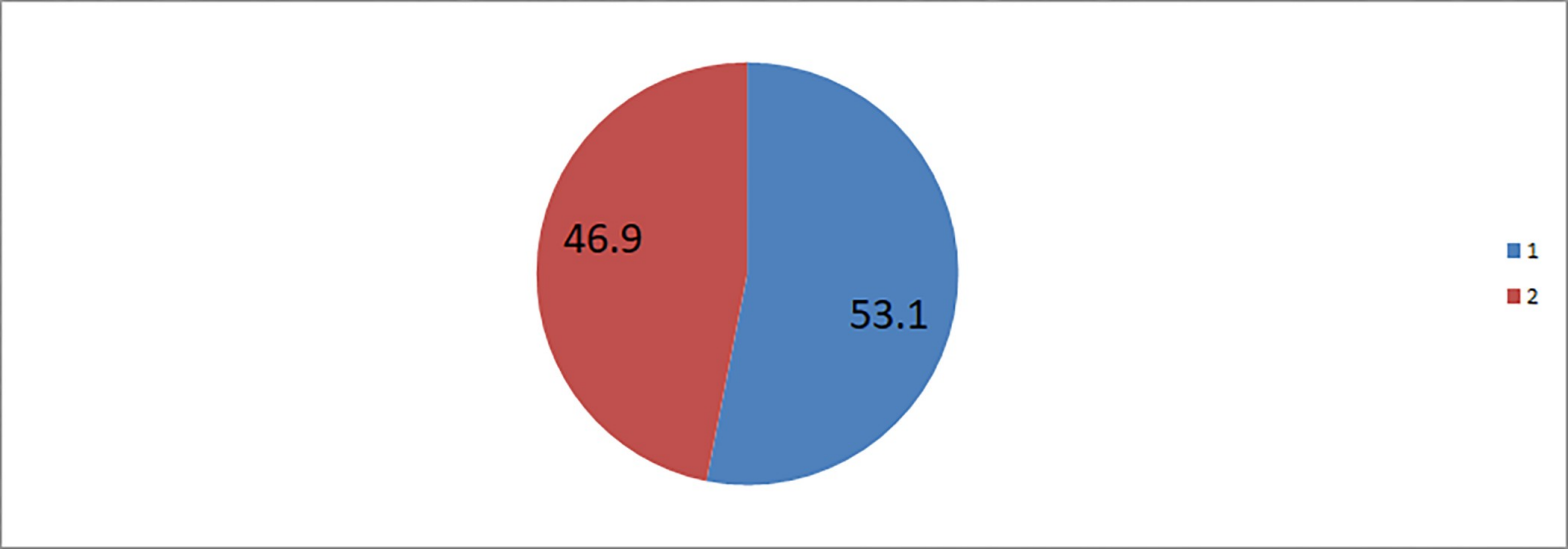
Knowledge on COVID-19 vaccination among pregnant women in Motta town and Hulet Eji Enese district northwest Ethiopia 2021. 1 = Inadequate knowledge on COVID-19 vaccination, 2 = Adequate knowledge on COVID-19 vaccination.

**Fig 2:**
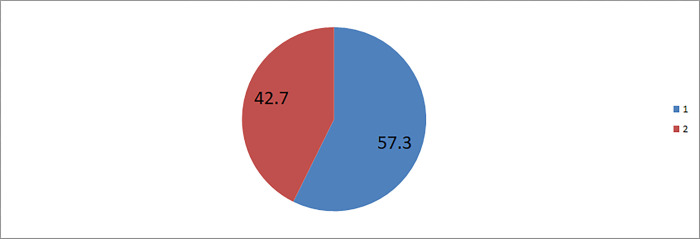
Attitude towards COVID-19 vaccination among pregnant women in Motta town and Hulet Eji Enese district, northwest Ethiopia, 2021. 1 = Negative attitude towards COVID-19 vaccination during pregnancy, 2 = Positive attitude Towards COVID-19 vaccination during pregnancy.

More than half 452 (53.1%) of study participants had inadequate knowledge on the presence of COVID-19 vaccination during pregnancy. Besides, 488(57.3%) of women had negative attitude towards COVID-19 vaccination during pregnancy “**Figs 1 and 2**”.

### Perception of risk regarding the use of COVID-19 vaccine among pregnant women

**Fig 3:**
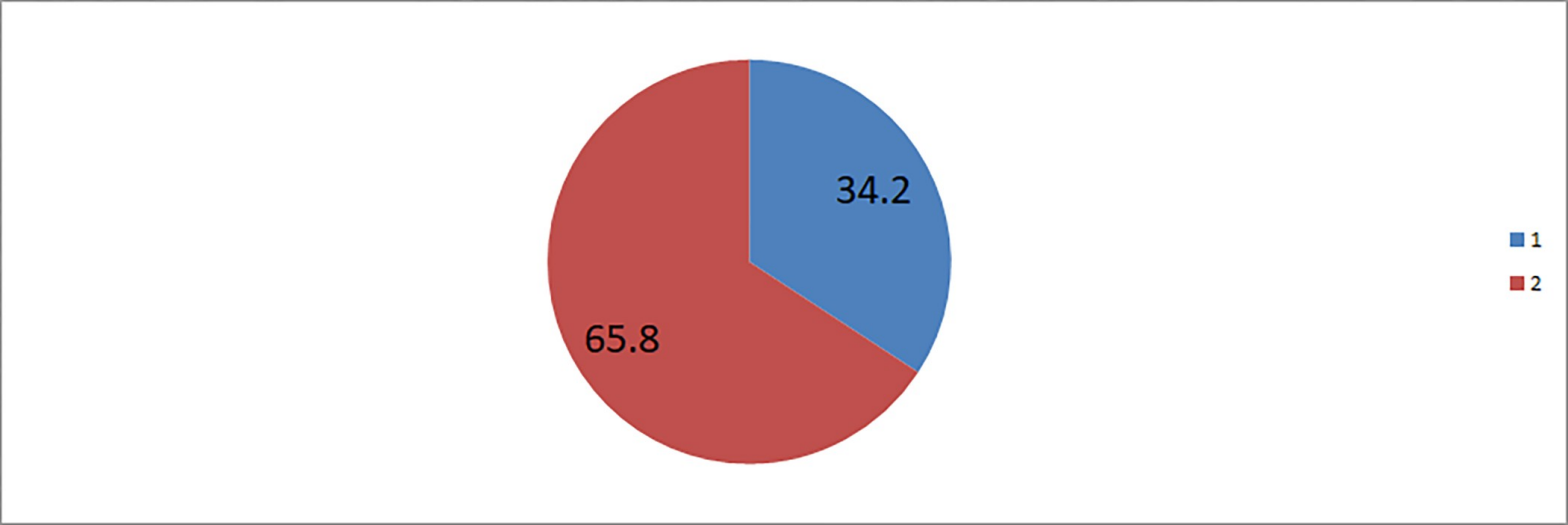
Perception of risk regarding the use of COVID-19 vaccine among pregnant women in Motta town and Hulet Eji Enese district, northwest Ethiopia, 2021. 1 = perception of not risk for pregnancy, 2 = perception of risk for pregnancy.

In this study the perception of risk regarding the use of COVID-19 vaccine during pregnancy was found to be 34.2% (95%CI: 31–37). The most common source of recommendation about the risk regarding the use of COVID-19 vaccination was family members (87.3%), and friends (11.7%) “**Fig 3**”.

### Factors associated with perception of risk regarding the use of COVID-19 vaccination during pregnancy

On bivariable logistic regression analysis residence, pregnancy type, ANC (**Antenatal Care)** service utilization, distance from the health facility, parity, educational level of woman, knowledge, and attitude towards COVID-19 vaccination had an association with perception of risk the use COVID-19 vaccination during pregnancy. However, on the multivariable logistic regression analysis unplanned pregnancy, had no used ANC service, long travel to reach the nearby health facility, lower educational level of women, and negative attitude towards COVID-19 vaccination were the factors that significantly associated with the perception of risk the use of COVID-19 vaccination during pregnancy.

In this study women with unplanned pregnancies were 3.66 times more likely to perceive as risk COVID-19 vaccination during pregnancy as compared to those women whose pregnancies were planned (AOR = 3.66; 95%CI: 2.31–5.81). Similarly, the odds of perception of risk COVID-19 vaccination during pregnancy were 7.07 times higher among women who hadn’t used ANC service compared with women who had used ANC service (AOR: 7.07; 95% CI: 4.35–11.5). This study also found that women who were traveling more than one hour to reach the nearby health facility were 4.57 times more likely to perceive as risk COVID-19 vaccination during pregnancy as compared to women who were traveling less than 30 minutes (AOR: 4.57; 95% CI: 2.34–8.91). likewise, women who had no formal education were 3.15 times more likely to perceive as risk COVID-19 vaccination during pregnancy as compared to women who had to attend college and above education (AOR: 3.15; 95%CI: 1.71–5.79). Besides, this study revealed that women who had secondary educational level were 5.18 times more likely to perceive as risk COVID-19 vaccination during pregnancy than those women attending college and above education (AOR: 5.18; 95% CI: 2.17–12.4). Lastly, the odds of perception of risk COVID-19 vaccination during pregnancy were 6.05 times higher among women who had a negative attitude towards COVID-19 vaccination (AOR: 6.05; 95%CI: 3.88–9.43) than women who had a positive attitude “**Table 3**”.

**Table 3:** Bivariable and multivariable logistic regression analysis of factors associated with the perception of risk COVID-19 vaccination during pregnancy, in Motta town and Hulet Eji Enese district, northwest Ethiopia, 2021.

Variables	Perception of risk		COR(95% CI)	AOR(95% CI)
	Yes No			
**Residence**				
Rural	276	452	4.4(2.5–7.7)	1.15(0.44–3.02)
Urban	15	108	1	1
**Wealth quintile**				
Lowest	70	99	1.96(1.24–3.11)	0.69(0.36–1.33)
Second	73	98	2.07(1.31–3.27)	1.07(0.57–2.01)
Middle	58	113	1.43(0.91–2.27)	1.08(0.56–2.08
Fourth	45	123	1.00(0.62–1.62)	0.6(0.31–1.17)
Highest	45	123	1	1
**Type of pregnancy**				
Planned	111	459	1	1
Unplanned	180	101	7.37(5.3–10.15)	3.66(2.31–5.81)***
**Distance from health facility**				
<30 minutes	31	282	1	1
30minutes to 1hr	64	139	4.19(2.61–6.73)	1.58(0.77–3.21)
>1hr	196	139	12.8(8.3–19.72)	4.57(2.34–8.91)***
**Antenatal care use**				
Yes	91	492	1	**1**
No	200	68	15.9(11.2–22.6)	7.1(4.35–11.5)***
**Parity**				
1–2	94	314	1	**1**
3–4	99	179	1.85(1.32–2.59)	0.87(0.51–1.49)
≥5	98	67	4.89(3.32–7.19)	0.72(0.38–1.37)
Educational level of the woman				
No formal education	233	338	2.71(1.5–4.9)	3.15(1.71–5.79)***
Grade1-8	41	94	1.72(0.87–3.37)	0.25(0.05–1.16)
Grade9-12	2	69	0.11(0.03–0.52)	5.18(2.17–12.4)***
College and above	15	59	1	**1**
**Knowledge on COVID-19 vaccin**				
Adequate	91	308	1	**1**
Inadequate	200	252	1.48(1.29–3.62)	1.34(0.88–2.05)
**Attitude towards COVID-19 vaccine**				
Positive attitude	61	427	1	**1**
Negative attitude	230	133	3.38(2.59–3.7)	6.05(3.8–9.43)***

AOR = Adjusted odd ratio, COR = Crude odd ratio, CI = Confidence interval * ^**^ **P ≤0.001.**

## Discussion

This community-based cross-sectional study assessed the perception of risk regarding the use of COVID-19 vaccination during pregnancy and associated factors among pregnant women in Motta town and Hulet Eji Enese district, northwest Ethiopia. More than one-third of mothers had a perceived risk of COVID-19 vaccination during pregnancy. Lower women’s educational level had not used antenatal care service, negative attitude towards COVID-19 vaccination, long travel time to get to the nearby health facilities, and unplanned pregnancy were significantly associated with perceived risk of COVID-19 vaccination during pregnancy.

In this study, the perception of risk regarding the use of COVID-19 vaccination during pregnancy is 34.2%.

The current study affirmed that unplanned pregnancy is significantly associated with the perception of risk regarding the use of COVID-19 vaccination during pregnancy. Thus women with unplanned pregnancies are 3.66 times more likely to perceive risk regarding the use of COVID-19 vaccination during pregnancy as compared to those women with a planned pregnancy. The possible explanation might be those women with unplanned pregnancies are more likely to be far from the scientific background and health care information’s about the prevention and control strategies of COVID-19 [19]. In addition to this, women with unplanned pregnancies, especially those unmarried women faced stigma from the community. To avoid the negative economic and social consequences of unplanned pregnancy, the only option in their mind is the termination of pregnancy. But still stigma of unplanned pregnancy doubled with abortion and they prefer to terminate in secret by using locally available herbal products rather than giving care by COVID-19 vaccination [16].

Antenatal care service utilization is another important predictor that is significantly associated with the perception of risk regarding the use of COVID-19 vaccination during pregnancy. Women who had no antenatal care visits were 7.07 times more likely to perceive the risk of COVID-19 vaccination during pregnancy as compared to women who had ANC visits. This might be those women who had no antenatal care follow-up have no opportunities to communicate with health care providers about the benefits and adverse outcomes of COVID-19 vaccination. Besides, those women having no antenatal care visits lack information and knowledge regarding pregnancy-related danger signs and actions that should be taken if such problems happened [20].

The present study revealed that long travel time to reach the nearby health facility is a significant factor that affects the perception of risk COVID-19 vaccination during pregnancy. Accordingly, women who travel more than one hour to reach the nearby health facility are 4.57 times more likely to perceive risk COVID-19 vaccination during pregnancy as compared to those women who travel less than thirty minutes. The possible explanation might be that long distances to reach the nearby health facility hinder women from accessing health care services and other information’s which is important for good health care-seeking practices. In addition, those women who travel longer, either have to expend extra costs for transportation or more time to access the modern health service [21].

The educational level of women is another factor that had significantly associated with the perception of risk COVID-19 vaccination during pregnancy. Thus, women who had no formal education and who had to attend secondary education were 3.15 and 5.18 times more likely to perceive risk COVID-19 vaccination during pregnancy as compared to those attending college and above respectively. This could be due to education being the main track for women’s employment, improved household economic status, and empowerment on decision making which in turn is associated with the use of modern maternal and child health services [22]. Women with better educational attainment will have good health literacy which in turn influences them to seek information and advice from health care providers on COVID-19 vaccination [23]. The reverse is true that women with lower educational status are more prone to traditional beliefs since they couldn’t stand alone and were even influenced by others’ decisions.

Lastly, the odds of perceived risk regarding the use of COVID-19 vaccination during pregnancy among pregnant women who had negative attitudes towards COVID-19 vaccination are 6.05 times more likely to perceive risk COVID-19 vaccination during pregnancy as compared to those mothers who had positive attitudes towards COVID-19 vaccination. This could be due to that attitude, satisfaction, and trust level plays a great role in influencing women’s choice of health care providers. Their attitude and perceptions towards COVID-19 vaccination triggers them to rely on it and to consider, free from life-threatening chemicals, no serious health implications, and lifelong effectiveness.

## Conclusions

Perception of risk COVID-19 vaccination during pregnancy was high. Long travel time to reach the nearest health facility, had no antenatal care service utilization, unplanned pregnancy, lower women’s educational level and negative attitude towards COVID-19 vaccination were factors significantly associated with perception of risk COVID-19 vaccination during pregnancy. Therefore, the government should design public health programs targeting the identified factor, and should minimize the perception of risk acquiring infection from CCOVID-19 vaccine to improve maternal and neonatal health outcome.

## Supporting information

S1 File (PDF)

S2 File (SAV)

## Abbreviations

ANC: Antenatal Care
AOR: Adjusted Odds Ratio
CI: Confidence Interval
COR: Crude Odds Ratio
ETB: Ethiopian Birr
SPSS: Statistical Package for Social Science
