# Supplementary material for: Perception of risk regarding the use of COVID-19 vaccine among pregnant women in Motta town and Hulet Eji Enese district, northwest Ethiopia
Source: PLoS One. 2022 Aug 24;17(8):e0269591. doi: 10.1371/journal.pone.0269591 (PMC9401104; doi:10.1371/journal.pone.0269591)
Supplement: S1 File — (PDF) [file pone.0269591.s001.pdf]

Date of interview (DD/MM/YYYY:___/_____/________

Sub city_ _____________________

Kebele No___________________

House No _____________________

**Part I. Socio-demographic information**		
101.	What is your age?	______________years
102.	Sex	1. Male<br>2. Female
103	What is your current marital status?	1. Married<br>2. Single<br>3. Divorced<br>4. Widowed<br>5. Separated
104	What is your religion?	1. Orthodox<br>2. Muslim<br>3. Protestant<br>4. Catholic<br>5. Other (specify)……….
105	What is your level of education?	1. Unable to read and write 4. Able to read and write<br>2. Grade 1-8 5. Grade 9-12<br>3. Certificate and diploma 6. Degree and above
106	What is your occupation?	1. Governmental employee 5. House wife<br>2. Private business 6. Student<br>3. Private employee 7. Daily laborer<br>4. Farmer 8. Others (specify)…………..
107	What is the estimated average household monthly income?	--------------------------------- ETH birr
108	What is the number of people in the household?	---------in number
PartII: **Maternal health care service and obstetrical related characteristics of participants**		
201	How many times have you had pregnant?	--------
202	Was your last pregnancy planned?	1. Yes<br>2. No
203	How many times have you had deliverd?	---------
204	Have you poor obstetric history before index baby?	1. Yes<br>2. No
205	If yes in Q no which problem?<br>More than one answers possible	1. Abortion<br>2. Intra uterine fetal death<br>3. Still birth<br>4. Early neonatal loss<br>5. Other specify ----------
206	Have you got antenatal care service?	1. Yes<br>2. No
207	How many times did you receive antenatal care?	-----
206	**Have you exposure to herbal medicines during pregnancy?**	1. Yes<br>2. No
207	**Distance from the nearby health facility**	1. Can be reached within 30 minutes<br>2. Takes 30 minutes to 1 hr<br>3. Takes more than 1 hr
**Part III a. Knowledge on Covid -19 virus**		
301	Where is the origin of COVID-19 pandemic	1. USA 3. China 4. Italy 5. Spain 6. I don't know

		2. Others (specify)-----
302	Can COVID-19 be transmitted from?	1. Person to person 3. From animals to person<br>2. From person animals 4. I don't know
303	What is the incubation period of Covid-19?	____________
304	What is/are the common symptoms of Covid-19? (**Multiple responses are possible)**	1. Fever 7.Sore throats 12. Loss of test<br>2. Headache 8. Chills 13. Loss of smell<br>3. Cough 9. Anorexia<br>4. Shortness of breath 10. Repeated shaking<br>5. Fatigue 11. Muscle pain (myalgia)<br>6. I don't know 14. Others (specify) ………
305	What is the recommended quarantine period for COVID-19 suspect?	_______________
306	Can asymptomatic persons with COVID-19 transmit the disease to other people?	1. Yes 2. No 3. Don't know
307	Whom do you think are at higher risk of Risk / developing sever disease from COVID-19? (Multiple responses are possible)	1. Cardiovascular disease 7. Diabetics 12. Hypertension<br>2. Chronic renal disease 8. Chronic respiratory diseases<br>3. Advanced age 9. Liver disease<br>4. Malignancy 10.Smokers 13. Male sex<br>5. Sever obesity 11. I don't know<br>6. Others (Specify)---------
308	What is/are the mode of COVID- transmission?	1. Respiratory droplet nuclei<br>2. Direct Contact (Hand shaking, kissing)<br>3. Indirect contact (Contaminated objects)<br>4. Airborne<br>5. Animal contacts<br>6. Mother to child<br>7. Don't know<br>8. Others (Specify---------)<br>9. I don't know
309	During which conditions will transmission of COVID -19 be higher?	1. Sneezing 4. Coughing 5. Singing 6. Talking<br>2. Others(specify)----------<br>3. I don't know

Part III b: Knowledge on COVID-19 prevention measures

310	Can early detection of COVID -19 help to prevent the disease transmission?	1. Yes 2.No 3. I don't know
311	Which of The following practices can help to protect from COVID-19? (Multiple responses are possible)	1. Washing hands with soap<br>2. Use of hand sanitizers<br>3. Covering nose with arms/ tissue during sneezing<br>4. Covering mouse with arms/ tissue during coughing<br>5. Wearing a face mask<br>6. Keeping physical distance<br>7. Staying at home<br>8. Avoid over crowding<br>9. Avoiding handshaking, hugging, or kissing<br>10. Other (specify)………………<br>11. I don't know
312	Are there specific vaccines to prevent COVID-19 as of today?	1. Yes 2. No 3. I don't know
313	How far one should stand/sit from a person to prevent contracting from COVID-19 infection?	_____________meter
314	Are there specific treatments that can cure COVID-19 as of today?	1. Yes 2. No 3.I don't know
315	What are the possible treatment options of COVID-19 as of today? (**Multiple responses are possible*)***	1. Anti-viral therapy 3. Vaccination<br>2. Supportive care 4.Others (specify-----)
316	Do pregnant women with suspected or confirmed Covid-19 need to give birth by cesarean section?	1. Yes 2. No 3.Don't know
317	Can women with Covid-19 breastfeed?	1. Yes 2. No 3. Don't know

Part IV a. Attitude towards Covid-19 virus

Instruction: The following Items are about attitude towards COVID-19. Please read each statement and circle a number 1, 2, 3, 4, 5

which indicates how much the statement applied to you. **1- Strongly disagree, 2-Disagree, 3-Neutral, 4- Agree, 5- Strongly agree**

S/N	Statement	1	2	3	4	5
401	Do you agree that COVID-19 will finally be successfully controlled?					
402	Do you agree that COVID-19 is a serious disease?					
403	Do you believe that COVID-19 can be treated at home					
404	Do you agree that, if anyone gets infected with COVID-19 has no any probability of recovering?					
405	Do you believe that you may get infected with COVID -19?					
406	Do you feel that older people and those with comorbid conditions are at higher risk of COVID -19 complications?					
407	Do you believe that most of our cultural values are jeopardized due to the preventive measures taken by the government?					
408	Do you agree that black people are less likely to be infected with COVID-19?					
**Part IV b: Attitude towards prevention measures of COVID 19 pandemic**						
409	Do you feel that you are well protected from COVID-19 while you have vaccinated ??					
410	Do you believe that Ethiopia can fully control the COVID-19 pandemic?					
411	Do you feel that avoiding touching eyes, nose, or mouth with hands can protect from contracting COVID-19?					
412	Do you agree that COVID-19 is preventable applying hand washing?					
413	Do you agree that applying physical distancing is important in controlling the spread COVID -19?					
414	Do you agree that applying staying at home is important in controlling the spread COVID -19					
415	Do you believe that the communities are complying with the recommended preventive measures?					
416	Will you accept to be quarantined if you get suspected of COVID -19?					
417	Will you accept to be isolated if you get infected of COVID -19 infection?					
**Part V. Preventive practice towards Covid-19 outbreak**						
501	Do you avoid vacations to prevent contracting and spreading COVID-19?	1. Yes 2.No				
502	Are you Appling the principle of home stay to protect contracting or spreading of COVID-19?	1. Yes 2.No				
503	If yes to Q "402", Are you spending your time by doing physical exercise?	1. Yes2. No				
504	Did you have any travel history out of your home town in the past 2 weeks?	1. Yes 2.No				
505	Did you wash your hands frequently with soap /hand sanitizers before and after contacting any one out of your home in the past 2 weeks??	1. Yes 2. No				
506	Have you been in any crowded place (crowded markets, churches etc.) in the past 2 weeks?	1. Yes 2. No				
507	Are you consistently using face masks when you are out of home in the past 2 weeks??	1. Yes 2.No				
508	Have you stand/sit 2 meters far away from a person in the past 2 weeks?	1. Yes 2. No				

Part VI: Perception risk COVID-19 vaccination during pregnancy

601	Being vaccinated for COVID-19 infection during pregnancy is a risk of contracting COVID-19 infection and adverse pregnancy outcome.	1. Yes<br>2. No
602	Level of risk of contracting COVID-19 infection	1. High risk<br>2. No risk<br>3. Risk similar to that of the general population

The End

Thank you
